# The Role of Job Satisfaction as a Mediator Between Professional Autonomy and Organizational Commitment Among Iranian ICU Nurses

**DOI:** 10.1155/jonm/6683020

**Published:** 2025-09-29

**Authors:** Naser Parizad, Yousef Mohammadpour, Vahid Alinejad, Aysan Judi

**Affiliations:** ^1^Patient Safety Research Center, Clinical Research Institute, Urmia University of Medical Sciences, Urmia, Iran; ^2^School of Nursing and Midwifery, Urmia University of Medical Sciences, Urmia, Iran; ^3^Department of Biostatistics, Urmia University of Medical Sciences, Urmia, Iran; ^4^Student Research Committee, Tabriz University of Medical Sciences, Tabriz, Iran

**Keywords:** intensive care units, job satisfaction, nurses, organizational commitment, professional autonomy

## Abstract

**Aims:**

This study examined the relationship between professional autonomy (PA) and organizational commitment (OC), with job satisfaction (JS) as a mediating factor among nurses employed in intensive care units (ICUs).

**Background:**

Previous research has established a link between nurses' PA and OC, but the mediating role of JS remains underexplored. Addressing this gap effectively is essential for improving nursing care outcomes and providing novel insights into how autonomy shapes commitment through JS.

**Methods:**

This descriptive-correlational study used structural equation modeling (SEM). A total of 420 ICU nurses were selected through quota sampling from Urmia educational hospitals in Iran from October 2022 to June 2023. Data were collected using a demographic survey questionnaire and Persian versions of the Minnesota Satisfaction Questionnaire (MSQ), Porter Organizational Commitment Questionnaire (P-OCQ), and Varjus Professional Autonomy Scale (V-PAS). Data were analyzed using SPSS version 23.0 and SmartPLS version 3.0.

**Results:**

Out of 420 participants, 385 fully completed the questionnaires (response rate: 92%). PA had a positive, weak direct association with OC (*β* = 0.273, *t* = 4.328, *p* < 0.001). It was strongly associated with JS (*β* = 0.846, *t* = 38.073, *p* < 0.001), which in turn was strongly associated with OC (*β* = 0.640, *t* = 10.515, *p* < 0.001). JS significantly mediated the association between PA and OC (*β* = 0.2477, 95% CI: 0.1956, 0.3000).

**Conclusion:**

PA was positively associated with OC among ICU nurses, with JS acting as a mediator. Enhancing both PA and JS is critical for improving nurses' commitment, leading to better quality care and patient safety.

**Implications for Nursing Management:**

Healthcare managers can empower nurses by involving them in patient care decision-making and supporting their independent decisions. Training to update nursing knowledge can expand their decision-making scope. Providing stress reduction programs, managing workloads, addressing nursing shortages, and offering financial and moral support can boost JS and OC.

## 1. Background

Professional autonomy (PA), a cornerstone of nursing identity, empowers nurses to make independent decisions based on their expertise to enhance patient care [1]. In nursing, PA refers to the freedom to act on professional knowledge and judgment to meet patients' needs, prioritizing quality care and patient well-being [2]. A recent integrative review confirmed that PA is critical for nurse retention and organizational commitment (OC), fostering loyalty and engagement with the workplace [3].

OC reflects nurses' loyalty, engagement, and desire to remain with their organization. It encompasses attitudinal commitment, which includes emotional attachment and willingness to adapt, as well as behavioral commitment, which signifies a strong bond to the organization [4]. Recent systematic reviews showed that high OC reduces turnover, enhances nurse–patient communication, improves patient safety, and elevates care quality [5–7]. OC is closely linked to job satisfaction (JS), defined as nurses' contentment with their work environment and role, which influences their quality of life, care quality, and patient satisfaction [8–10]. Theoretically, JS mediates the relationship between PA and OC, as autonomy fosters a positive work experience that enhances satisfaction, subsequently strengthening OC [11, 12]. This mediation model is grounded in motivational theories, such as self-determination theory, which posits that autonomy satisfies intrinsic needs, leading to greater JS and OC [13].

Nurses play a critical role in patient safety and societal health, particularly in high-stake environments like intensive care units (ICUs). ICU nurses face unique challenges, including high-acuity patients, inadequate nurse staffing, complex decision-making, and intense workloads, which amplify the importance of PA and its impact on JS and OC [11, 14]. Despite advanced qualifications (e.g., master's degrees or specialized training), ICU nurses may not experience high JS due to demanding work conditions, making this an ideal context for studying these relationships [15]. Although prior studies have confirmed a direct link between PA and OC, the mediating role of JS remains underexplored in ICUs. This research aimed to address this gap by examining how JS mediates the relationship between PA and OC among ICU nurses. Given the potential bidirectional nature of these relationships, this study also examined a reverse model to investigate whether OC and JS contribute to increased PA. Understanding these reciprocal dynamics can provide valuable insights for nursing managers seeking to strengthen PA, JS, and OC. Such efforts are significant in ICU settings, where improving work conditions can lead to better outcomes for nurses, patients, and the broader healthcare system, especially in resource-constrained environments like Iran.

### 1.1. Theoretical Framework and Hypothesis Development

The study was grounded in self-determination theory [13], which posits that autonomy enhances intrinsic motivation, JS, and OC. Self-determination theory explains how PA influences nurses' outcomes in high-stake environments like ICUs. Empowerment theory [16] further supports that autonomy and supportive environments enhance psychological empowerment, improving JS and OC. This section included international literature from various cultural contexts to strengthen the generalizability of findings from Iran and Asia.

### 1.2. PA and OC

Research has consistently demonstrated a positive association between PA and OC across various cultural contexts. In Europe, Zychová et al. [12] found that PA significantly predicted both JS and OC among healthcare professionals in the Czech Republic [12]. Similarly, in a French study, Gillet et al. [9] reported that autonomy-supportive environments enhanced nurses' commitment and quality of care in oncology settings, emphasizing the importance of autonomy in individualistic cultures where professional agency is highly valued [9]. In the United States, Spence Laschinger et al. [17] found that structural empowerment, including access to autonomy, significantly increased OC among hospital nurses, suggesting that autonomy fosters a sense of organizational loyalty in North American healthcare settings [17]. In Australia, Talukder [18] confirmed a positive association between workplace autonomy and OC, mediated by JS and work–life balance, in a context where individual agency is culturally prioritized [18]. These findings align with studies from Asian contexts, such as Kim and Kim [15], who reported a similar link in South Korean hospitals, and Judi et al. [11], who found that PA and OC improved service quality and patient safety in Iranian ICUs [11, 15]. The consistency across these diverse settings—ranging from individualistic Western cultures to collectivist Asian ones—supports the universal importance of PA in enhancing OC. Thus, we hypothesized the following:

  H1: PA is positively associated with OC among nurses.

### 1.3. The Mediating Role of JS

A strong positive association between PA and JS has been established across global healthcare settings. In the Czech Republic, Zychová et al. [12] found that autonomy significantly increased JS among employees, enhancing their retention and organizational performance [12]. Similarly, in a Scandinavian context, Pursio et al. [3] conducted an integrative review across multiple Western countries, concluding that PA is a critical driver of JS, particularly in environments that support independent decision-making [3]. In South Korea, Kim et al. [19] reported that greater PA increased nurses' JS, particularly when acting as physician assistants, highlighting the role of autonomy in high-responsibility roles [19]. Higher JS, in turn, fosters enthusiasm, loyalty, and OC, as demonstrated in a Jordanian study by Otoum et al. [20], which found that JS mediates the relationship between job performance and OC [20]. In a U.S.-based meta-analysis, Xu et al. [21] confirms a moderate to strong relationship between JS and OC, emphasizing its mediating role across professional settings [21]. These findings are consistent with Iranian studies, such as Judi et al. [11], which reports a strong JS-OC link in healthcare [11]. The global evidence supports the mediating role of JS, particularly in individualistic cultures where autonomy directly enhances personal satisfaction, and in collectivist settings where it fosters group-oriented commitment. Therefore, we hypothesized the following:

  H2: PA is positively associated with JS among nurses.
  H3: JS is positively associated with OC among nurses.
  Core hypothesis: JS mediates the association between PA and OC among nurses.

### 1.4. OC and PA (Mediating Role of JS)

The bidirectional relationship between OC and PA, with JS as a mediator, is less explored but increasingly relevant. In a study conducted in Saudi Arabia, Rawah and Banakhar [22] found that high OC fosters environments where nurses felt trusted and empowered, leading to greater PA [22]. Similarly, in a Taiwanese study, Huang et al. [23] reported that OC and JS may also strengthen PA by fostering organizational support and trust, particularly in collaborative acute care settings [23]. These findings align with those of Judi et al. [11], who noted that higher OC is associated with increased PA among Iranian ICU nurses, suggesting that committed nurses are more likely to be granted autonomy [11]. In a cross-national study involving 20 European countries, Suzuki and Hur [24] found that OC enhances perceived autonomy through increased trust and empowerment, particularly in bureaucratic structures [24]. The relationship between OC and JS is well-documented globally, with studies like Ebraze et al. [25] in Iran and Ćulibrk et al. [8] in Serbia confirming that committed employees report higher JS due to alignment with organizational goals [8, 25]. Furthermore, JS has been shown to enhance PA, as satisfied nurses feel more confident in exercising professional judgment, as evidenced in a Finnish study by Pursio et al. [3, 3]. These international findings suggest that OC and JS create a supportive environment that enhances PA, particularly in settings that value empowerment and trust. Thus, we hypothesized the following:

  H4: OC is positively associated with PA among nurses.
  H5: OC is positively associated with JS among nurses.
  H6: JS is positively associated with PA among nurses.
  Exploratory hypothesis: JS mediates the association between OC and PA among nurses.

**Figure 1:**
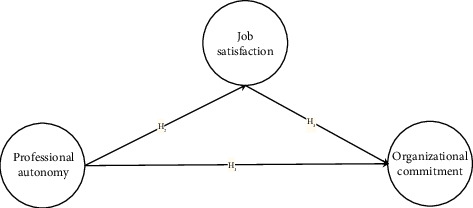
Conceptual framework of the hypothesized direct model. The figure shows the hypothesized direct model where job satisfaction mediates the relationship between professional autonomy and organizational commitment.

**Figure 2:**
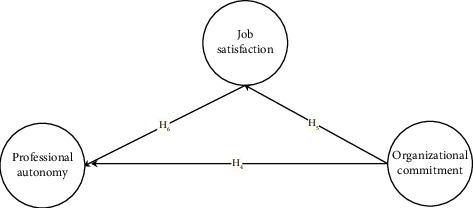
Conceptual framework of the hypothesized reverse model. The figure shows the hypothesized reverse model where job satisfaction mediates the relationship between organizational commitment and professional autonomy.

The conceptual framework visually represents the study variables and their expected relationships as shown in Figures 1 and 2.

## 2. Methods

### 2.1. Study Design and Sampling

This cross-sectional, descriptive-correlational study utilized structural equation modeling (SEM). The study population consisted of nurses working in ICUs of Urmia educational hospitals in Iran.

The sample size was determined using a sample-to-item ratio criterion, which required a minimum ratio of 5:1 to ensure sufficient statistical power for SEM [26]. The study employed 52 items across three validated instruments: the Varjus Professional Autonomy Scale (V-PAS, 18 items), the Porter Organizational Commitment Questionnaire (P-OCQ, 15 items), and the Minnesota Satisfaction Questionnaire (MSQ, 19 items). To meet the 5:1 ratio, 420 ICU nurses were recruited, yielding an 8:1 ratio, which supported robust path analysis and mediation testing. The sample size adequacy for the study was assessed using G∗Power software. Assuming a minimum correlation coefficient of 0.15 for detecting a statistically significant relationship between variables [27], a sample size of 350 participants was required to achieve 95% confidence and 80% statistical power. Considering a possible 20% attrition rate, the final sample size was set at 420 nurses. Ultimately, 35 incomplete questionnaires were excluded from the final analysis.

A quota sampling approach was employed, with proportional distribution according to the number of ICU nurses (total: 503) across four hospitals. Initially, the sampling ratio (420/503 = 0.835) was used to determine the number of participants per hospital (e.g., Imam Khomeini Hospital: 220 × 0.835 = 184). The same approach was applied to calculate the sample sizes for other hospitals (Table 1).

Inclusion criteria were full-time ICU nurses with a bachelor's degree or higher, at least 6 months of work experience, and willingness to participate. Nurses on annual, extended, or unpaid medical leave were excluded, as were those submitting questionnaires with > 20% missing responses. The six-month minimum work experience criterion was selected based on Benner [28] “From Novice to Expert” model, which suggests that nurses transition from novice to advanced beginner within 6–12 months, gaining sufficient experience to form stable professional perceptions [28]. This criterion is further supported by Van Maanen and Schein [29] organizational socialization theory, which indicates that the initial 6–12 months are critical for internalizing workplace norms and values, enabling nurses to develop reliable assessments of their autonomy, satisfaction, and commitment [29]. By including only nurses with at least 6 months of ICU experience, the study ensured that participants were beyond the initial adjustment phase, enhancing the validity of their questionnaire responses.

### 2.2. Outcome Measures

Data were collected using a demographic survey questionnaire and Persian versions of the P-OCQ, V-PAS, and MSQ. All instruments were translated and culturally validated for the Iranian ICU context following established guidelines for crosscultural adaptation [30]. The translation process involved forward translation by two bilingual experts, reconciliation, back-translation by an independent translator, and review by a panel of five nursing faculty members to ensure linguistic and cultural equivalence. Content validity was confirmed with Item-level Content Validity Index (I-CVI) scores of 0.80–1.00.

I. The demographic survey questionnaire included age, gender, marital status, education level, employment status, the name of the hospital, the type of units, and work experience.
II. P-OCQ
  The 15-item questionnaire, developed by Mowday et al., measures OC using a seven-point Likert scale, ranging from “*strongly disagree* = 1” to “*strongly agree* = 7.” The total score ranges from 15 to 105. Scores ranging from 70 to 105 indicate high OC, scores between 35 and 70 show moderate OC, and scores below 35 suggest low OC. Items 3, 7, 9, 11, 12, and 15 are reverse-scored. The original P-OCQ had a Cronbach's alpha of 0.88 [31]. In Australia, reliability was confirmed with a Cronbach's alpha of 0.89 [18]. In Iran, Seyyedmoharrami et al. [32] validated the Persian version with faculty members' review and reported a Cronbach's alpha of 0.80 [32]. In this study, the Persian P-OCQ's reliability was confirmed with a Cronbach's alpha of 0.82.
III. V-PAS
  The 18-item questionnaire, developed by Varjus et al. [33], assesses ICU nurses' autonomy across three domains: action-, knowledge-, and value-based autonomy. Items are evaluated using a six-point Likert scale that ranges from “*strongly disagree* = 1” to “*strongly agree* = 6.” The scoring scale ranges from 18 to 108 (18–47.99 = low autonomy, 48–77.99 = moderate autonomy, and 78–108 = high autonomy). The original V-PAS showed Cronbach's alpha values of 0.56 (knowledge), 0.62 (action), and 0.76 (values) [33]. In Iran, Yeganeh et al. [34] validated the Persian V-PAS, reporting a Cronbach's alpha of 0.99 [34]. In this study, a pilot test with 30 nurses using a test-retest approach over two weeks yielded a reliability of 0.88. Cultural validation involved expert panel review, ensuring relevance to Iranian ICU settings.
IV. MSQ
  The 20-item questionnaire, developed by Weiss et al. [35], assesses JS through intrinsic (items 1, 2, 3, 4, 7, 8, 9, 10, 11, 15, 16, 20) and extrinsic (items 5, 6, 12, 13, 14, 19) subscales, using a 5-point Likert scale from “*very satisfied* = 5” to “*very dissatisfied* = 1.” The total score ranges from 20 to 100 (≤ 25 = low JS, 26–74 = moderate JS, ≥ 75 = high JS) [35]. The short form was chosen for its established validity and efficiency in reducing respondent burden in high-stress ICU environments, without compromising psychometric integrity. The MSQ had a Cronbach's alpha of 0.89 in Poland [36] and 0.82 for the Persian version in Iran [37]. In this study, the Persian MSQ's Cronbach's alpha was 0.85. Cultural validation ensured applicability to Iranian ICU nurses through expert panel review and pilot testing.

### 2.3. Data Collection

The researcher obtained permission from the Research Deputy and approval from the Institutional Review Board of Urmia University of Medical Sciences. The lead researcher visited ICU departments, obtained participant lists from hospital authorities, and distributed anonymous paper-based questionnaires to participants. Over three shifts (day, evening, and night) from Saturday to Friday, the researcher explained the study's objectives, obtained written consent, and allowed nurses sufficient time to complete questionnaires independently. Participants were assured that their information would remain confidential. Completed questionnaires were collected in sealed envelopes and stored in a locked cabinet at the research office of Urmia University of Medical Sciences, accessible only to the lead researcher and authorized study personnel. Data were entered into a password-protected electronic database, with all identifiable information removed to ensure anonymity. Data were collected from October 2022 to June 2023.

### 2.4. Data Analysis

Data were analyzed using SPSS version 23 (IBM Corp., Armonk, N.Y., USA) and SmartPLS software version 3.0 [38]. Quantitative variables were reported as the mean ± standard deviation, and qualitative variables were presented as numbers (percentages). The Fornell–Larcker criterion was used to examine the model's discriminant validity. Cronbach's alpha coefficient was employed to verify the model's reliability. Path coefficients (*β*) and t-values were used to assess the primary SEM model. The macro bootstrap method was applied to test the mediation effects. A *p* value < 0.05 was considered statistically significant.

### 2.5. Ethical Approval

This manuscript, part of a nursing thesis, adheres to the Declaration of Helsinki. Previous findings on PA, JS, OC, and job performance among Iranian ICU nurses (4 hospitals) were published elsewhere [11]. Approval was obtained from the Research Review Board of Urmia University of Medical Sciences (Date: 03/08/2023/No: IR.UMSU.REC.1401.418). All the participants provided written informed consent.

## 3. Results

Of 420 distributed questionnaires, 385 were completed (response rate: 92%). After excluding incomplete responses, the final sample comprised 385 nurses, maintaining adequate power for the SEM analysis. Participants had a mean age of 38.19 years (SD = 7.22) and mean work experience of 13.34 years (SD = 6.79), including 7.54 years (SD = 5.12) in ICUs. Most nurses were married (66.8%, *n* = 257), female (57.4%, *n* = 221), and held bachelor's degrees (74.8%, *n* = 288). Shift work distribution was 43.9% (*n* = 169) on day shifts, 5.2% (*n* = 20) on evening shifts, and 50.9% (*n* = 196) on rotating shifts. Employment status included 51.2% (*n* = 197) permanent, 28.3% (*n* = 109) probationary, and 20.5% (*n* = 79) contractual. Unit distribution was 42.1% (*n* = 162) in other units, 36.6% (*n* = 141) in general units, 17.1% (*n* = 66) in cardiac units, and 4.2% (*n* = 16) in trauma units.

Mean scores for PA, JS, and OC, alongside Cronbach's alpha coefficients (> 0.7, indicating good reliability) are presented in Table 2. The results indicated that most nurses exhibited moderate levels of PA, JS, and OC (Table 3). The model also met the Fornell–Larcker criterion, ensuring acceptable discriminant validity (Table 4).

### 3.1. General Conceptual Model

**Figure 3:**
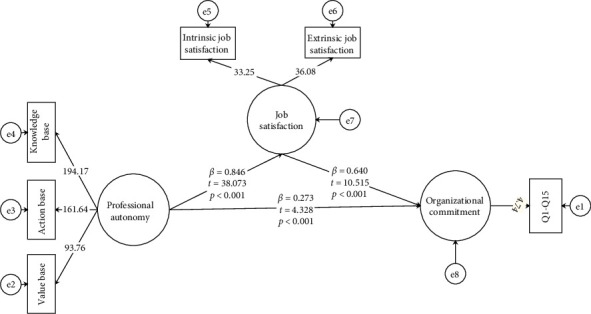
The direct model (*N* = 385). Structural equation model for the general conceptual model showing relationships among professional autonomy (PA), job satisfaction (JS), and organizational commitment (OC). Note: path coefficients (*β*) represent the standardized strength and direction of the relationship between variables. *T*-values assess the statistical significance of the path coefficients, with *t* > 1.96 indicating significance at *p* < 0.05. Significance levels (*p*) denote the probability of the observed relationship occurring by chance, with *p* < 0.05 considered statistically significant.

SEM revealed significant relationships among the variables. PA had a weak positive direct relationship with OC (*β* = 0.273, *t* = 4.328, *p* < 0.001), indicating a statistically significant but modest relationship. In contrast, PA demonstrated a strong positive relationship with JS (*β* = 0.846, *t* = 38.073, *p* < 0.001). JS had a strong relationship with OC (*β* = 0.640, *t* = 10.515, *p* < 0.001) (Table 5, Figure 3).

All t-values exceeded 1.96, indicating significance at a 95% confidence level. Bootstrap analysis confirmed JS as a significant mediator between PA and OC (indirect effect: *β* = 0.2477, 95% CI: 0.1956, 0.3000) (Table 6).

Model fit was evaluated using established indices following methodological standards [39, 40]. The fit indices were *χ*^2^ = 2486.68, RMSEA = 0.093, PNFI = 0.93, NNFI = 0.99, NFI = 0.97, IFI = 0.99, CFI = 0.99, SRMR = 0.05, RFI = 0.97, and rms Theta = 0.078. According to Hu and Bentler [39], a good model fit is generally indicated by RMSEA ≤ 0.06, SRMR ≤ 0.08, and CFI, NFI, and IFI ≥ 0.95. While most indices in our model met the recommended thresholds, the RMSEA value of 0.093 exceeded the conventional cut-off for good fit and falls within the range of marginal or mediocre fit (0.08–0.10) as suggested by Browne [41]. This implies that while the model demonstrated an acceptable representation of the data, it may not fully capture all underlying constructs or complexities, possibly due to contextual factors or omitted variables.

### 3.2. General Conceptual Reverse Model

**Figure 4:**
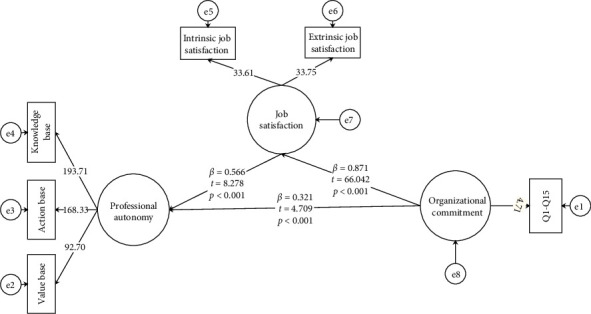
The reverse model (*N* = 385). Structural equation model for the reverse conceptual model showing relationships among organizational commitment (OC), job satisfaction (JS), and professional autonomy (PA). Note: path coefficients (*β*) represent the standardized strength and direction of the relationship between variables. *T*-values assess the statistical significance of the path coefficients, with *t* > 1.96 indicating significance at *p* < 0.05. Significance levels (*p*) denote the probability of the observed relationship occurring by chance, with *p* < 0.05 considered statistically significant.

To explore bidirectional relationships, as hypothesized in the introduction and detailed in the methods, a reverse model was tested to assess whether OC and JS were related to PA. Results showed OC had a moderate positive relationship with PA (*β* = 0.321, *t* = 4.709, *p* < 0.001) and a strong positive relationship with JS (*β* = 0.871, *t* = 66.042, *p* < 0.001). Furthermore, JS showed a moderate positive relationship with PA (*β* = 0.566, *t* = 8.278, *p* < 0.001) (Table 7, Figure 4).

All t-values were above 1.96, confirming significance at a 95% confidence level. Bootstrap analysis revealed a significant indirect relationship between OC and PA through JS (indirect effect: *β* = 0.2744, 95% CI: 0.1122, 0.4306) (Table 8), though the inclusion of zero in the confidence interval suggested caution in interpreting the mediation's strength.

Fit indices for the reverse model were as follows: *χ*^2^ = 2242.93, RMSEA = 0.099, PNFI = 0.91, NNFI = 0.92, NFI = 0.90, IFI = 0.92, CFI = 0.88, SRMR = 0.09, RFI = 0.91, and rms Theta = 0.069. Compared to the original model, the reverse model showed poorer fit, with RMSEA (0.099) approaching the upper limit of marginal acceptability (0.08–0.10) and CFI (0.88) falling below the recommended threshold of 0.95 [39, 40]. The SRMR (0.09) slightly exceeded the optimal cut-off of 0.08, further indicating a less robust fit. These results indicated that the reverse model was less well-supported, potentially due to unmodeled contextual factors or the complexity of bidirectional relationships in this setting.

Model comparison using AIC (original: 115; reverse: 120) and BIC (original: 130; reverse: 133) confirmed a better fit for the original model, supporting the hypothesized directional relationships [42, 43]. The goodness-of-fit (GoF) indices further corroborated the original model's superiority.

## 4. Discussion

This study aimed to evaluate the relationship between PA and OC among ICU nurses, with JS as a mediator. The findings confirm that PA has a positive relationship with OC, with higher autonomy associated with greater commitment. These results align with prior studies, such as Labrague et al. [44] who found a positive link between PA and OC in Philippine hospitals [44] and Suzuki and Hur [24] who reported similar associations [24]. Judi et al. [11] emphasized that higher autonomy and greater commitment enhance patient safety and care quality in nursing [11].

However, the nature and strength of these relationships may vary across different cultural contexts. In Western individualistic societies—such as the U.S., the U.K., and Scandinavian countries—autonomy is often seen as a core professional right and strongly tied to personal JS and commitment [3, 45]. Studies conducted in these contexts reported more substantial direct effects of PA on OC [3, 12, 44], likely reflecting the higher value placed on individual agency. For example, Gillet et al. [9] in France found that PA is a major predictor of both commitment and retention in oncology nurses [9]. In a Czech study, Zychová et al. [12] reported that PA significantly contributes to JS and organizational loyalty across various healthcare settings [12]. In an Australian context, Talukder [18] highlighted that autonomy indirectly enhances commitment via satisfaction and reduced work–family conflict [18]. These findings suggest that in more individualistic cultures, autonomy may have a more prominent direct effect on organizational outcomes.

In contrast, Iranian ICUs operate within a more collectivist cultural framework and a hierarchical clinical structure, where physician-led decision-making is a standard practice. This may limit the practical expression of autonomy, reducing its direct influence on commitment and increasing the importance of contextual factors such as JS. This cultural lens helps explain the relatively weaker direct effect of PA on OC found in our model.

This study also revealed that JS significantly mediated the relationship between PA and OC. Higher autonomy was associated with higher JS, which is linked to greater OC, consistent with self-determination theory [13]. To better understand this mediating pathway, we examined the underlying components of JS as measured by the MSQ. The scale distinguishes between intrinsic factors (e.g., autonomy, responsibility, and skill use) and extrinsic factors (e.g., pay, supervision, and working conditions). While our study did not test these dimensions separately, prior research suggests that intrinsic factors tend to have a stronger impact on OC in professional roles such as nursing [9, 46]. It is likely that autonomy and professional growth opportunities, as intrinsic motivators, play a key role in reinforcing commitment through enhanced JS among ICU nurses. This mediation is supported by studies showing that JS reduces stress and burnout, thereby enhancing commitment [46, 47]. Otoum et al. [20] further confirmed JS's mediating role between OC components and job performance [20]. No contradictory findings have been identified, indicating robust support for the mediation model.

The reverse model demonstrated significant bidirectional associations, with higher OC and JS associated with greater PA. This finding supports the notion that autonomy is not only a cause but also a consequence of positive organizational experiences. Committed and satisfied nurses may feel more empowered and trusted, which in turn enhances their perceived autonomy. This dynamic aligns with self-determination theory, which emphasizes the reciprocal relationship between supportive environments and the internalization of autonomous motivation [13]. It also echoes empowerment frameworks, where OC fosters psychological empowerment through perceived competence, meaning, and impact [16]. Thus, rather than being a post hoc exploration, the reverse model offers a theoretically grounded extension of our main model, highlighting the complex, bidirectional dynamics among PA, JS, and OC in ICU nursing practice.

These culturally embedded and psychologically complex relationships suggest that strategies to enhance ICU nurses' commitment should not rely solely on increasing autonomy. Instead, they must also focus on creating supportive, intrinsically rewarding environments that foster JS and recognition, thereby strengthening both OC and perceived autonomy in a reinforcing cycle. This aligns with self-determination theory, which posits that fulfilling intrinsic needs for autonomy, competence, and relatedness enhances motivation and commitment in high-stress environments like ICUs [13]. Similarly, psychological empowerment theory emphasizes that environments fostering meaning and self-determination create a virtuous cycle of engagement and autonomy, particularly in collectivist settings where organizational support is critical [16].

## 5. Limitations

This study has several limitations. First, its timing during the COVID-19 pandemic affected data collection among ICU nurses. Limited access and increased workloads made questionnaire distribution challenging. To facilitate participation and maintain anonymity, nurses were allowed to complete surveys at flexible times; however, this may have produced varied responses due to differences in stress levels and resource availability. Future research in diverse hospital settings could help assess the generalizability of these findings. Second, the results are specific to ICU nurses in Iranian educational hospitals, which limits their applicability to other departments, hospitals, or cultural contexts. The large number of items across the three questionnaires may also have reduced nurses' willingness to respond, given their heavy workloads. To address this, surveys were completed anonymously, in a calm environment, and at participants' own pace. Third, the study did not statistically control for potential moderating effects of variables such as age, shift type, and ICU work experience in the structural model, even though these factors were included in the demographic analysis. Their exclusion limits the ability to isolate the unique effects of PA on JS and OC. Future studies should use covariate-adjusted models or multigroup comparisons to improve internal validity. Fourth, despite a high response rate of 92%, a slight risk of nonresponse bias remains. However, comparisons between early and late respondents showed no significant differences in key variables, suggesting minimal selection bias. Quotas were carefully managed to reduce sampling errors associated with nonprobability quota sampling. Fifth, the cross-sectional design limits causal inference and prevents determining the temporal sequence of relationships among PA, JS, and OC, particularly regarding the bidirectional effects found in the reverse model. Finally, the RMSEA values for the main model (0.093) and reverse model (0.099) fall within the marginal fit range. According to Browne [41], RMSEA values between 0.08 and 0.10 indicate mediocre fit [41]. Although other indices suggested good model fit, these marginal RMSEA values warrant caution in interpreting the model's adequacy. Such limitations may stem from contextual factors in Iranian ICU settings or unmeasured confounders. Future research should improve model specification and expand measurement indicators to enhance overall fit.

## 6. Conclusion

PA was positively associated with nurses' OC, with JS as a mediator in this association. JS is vital for enhancing nurses' commitment, leading to improved care quality and patient safety. Organizations should prioritize strategies to increase PA and JS to strengthen OC. Future research should investigate the long-term effects of PA on JS and OC in various hospital settings and healthcare systems. Experimental or longitudinal designs could help confirm the causal relationships suggested by the current SEM models. Additionally, qualitative studies could reveal the mechanisms linking these factors, particularly in high-stress environments such as ICUs. Future studies are encouraged to explore whether key sociodemographic factors (e.g., age, shift type, and ICU experience) moderate the relationships among PA, JS, and OC. This could help identify specific groups of nurses who may benefit most from targeted interventions to enhance their autonomy and engagement.

## 7. The Implication for Nursing Management

Healthcare managers can enhance ICU nurses' PA, JS, and OC by implementing targeted strategies. Establishing structured shared governance models, like unit-based councils, allows nurses to participate in decision-making and policy development [44]. Supporting independent clinical judgments in critical situations empowers nurses, improving care quality [48].

Multifaceted interventions, including peer support groups and mental health resources, can mitigate burnout and boost JS [46]. Addressing staffing shortages through competitive salaries and recognition programs can alleviate workload pressures. Clear communication of organizational goals and performance-based rewards can further enhance OC and PA [13]. These actions can reduce turnover and improve patient outcomes in ICUs.

## Figures and Tables

**Table 1:** Number of nurses by hospital.

Hospital name	Number of nurses in ICU	Number of nurses in study
Imam Khomeini Hospital	220	184
Taleghani Hospital	130	108
Shaheed Motahari Hospital	74	62
Seyed Al-Shohada Hospital	79	66
Total	503	420

*Note:* Quota sampling was used to proportionally distribute the sample (*N* = 420) based on the total number of ICU nurses (*N* = 503) across four hospitals.

**Table 2:** Study variables' mean and standard deviation.

Variables	*N*	Mean	Standard deviation	Cronbach's alpha
Professional autonomy	385	63.57	16.62	0.958
Job satisfaction	385	56.39	11.54	0.898
Organizational commitment	385	27.77	8.2	0.872

*Note:* Professional autonomy (V-PAS, score range: 18–108); Job Satisfaction (MSQ, score range: 20–100); organizational commitment (P-OCQ, score range: 15–105). Cronbach's alpha: measure of internal consistency reliability (values > 0.7 indicate good reliability). *N* = 385 (number of ICU nurses included in the analysis after excluding incomplete responses).

**Table 3:** Frequency distribution of variables.

Variables		*N*	%
Professional autonomy	High	86	22.30
	Moderate	228	59.20
	Low	71	18.40

Job satisfaction	High	178	46.20
	Moderate	189	49.10
	Low	9	2.30

Organizational commitment	High	123	31.90
	Moderate	259	67.30
	Low	3	0.80

*Note:* Professional autonomy (low: 18–47.99, moderate: 48–77.99, and high: 78–108); job satisfaction (low: ≤ 25, moderate: 26–74, high: ≥ 75); organizational commitment (low: < 35, moderate: 35–70, high: 70–105). Percentages may not sum to 100% due to rounding.

**Table 4:** Results of Fornell and Larcker's method for assessing discriminant validity.

Variables	Professional autonomy	Organizational commitment	Job satisfaction
Professional autonomy	1		
Organizational commitment	0.815	1	
Job satisfaction	0.854	0.873	1

*Note:* Values represent correlation coefficients assessed using the Fornell–Larcker criterion to establish discriminant validity. Diagonal values are the square root of the average variance extracted (AVE) for each construct.

**Table 5:** Results of structural equation analysis for the general conceptual model.

Path	Beta coefficient (*β*)	*t*-value	Significance (*p*)	Result
H1: PA ⟶ OC	0.273	4.328	< 0.001	Confirmed
H2: PA ⟶ JS	0.846	38.073	< 0.001	Confirmed
H3: JS ⟶ OC	0.640	10.515	< 0.001	Confirmed

*Note: N* = 385 (number of ICU nurses included in the analysis after excluding incomplete responses). PA: Professional autonomy, measured by the Varjus Professional Autonomy Scale (V-PAS). JS: Job satisfaction, measured by the Minnesota Satisfaction Questionnaire (MSQ). OC: Organizational commitment, measured by the Porter Organizational Commitment Questionnaire (P-OCQ). Beta coefficient (*β*): standardized path coefficient indicating the strength and direction of the relationship between variables. *t*-value: Test statistic for assessing the significance of the path coefficient (*t* > 1.96 indicates significance at *p* < 0.05). Significance (*p*): probability value indicating statistical significance (*p* < 0.05 considered significant).

**Table 6:** Indirect effects and bootstrapping results with mediation analysis for the main model.

Path	Indirect effect	Bootstrap standard errors	95% Bootstrapped confidence interval	
			Lower	Upper
Professional autonomy to organizational commitment through job satisfaction	0.2477	0.0267	0.1956	0.3000

*Note:* Indirect effect: the effect of the independent variable on the dependent variable through the mediator (job satisfaction). Bootstrap standard errors and 95% bootstrapped confidence interval: derived from 5000 bootstrap samples to assess the significance of the mediation effect. Confidence intervals excluding zero indicate significant mediation.

**Table 7:** Results of structural equation analysis for the reverse conceptual model.

Path	Beta coefficient (*β*)	*t*-value	Significance (*p*)	Result
H1: OC ⟶ PA	0.321	4.709	< 0.001	Confirmed
H2: OC ⟶ JS	0.871	66.042	< 0.001	Confirmed
H3: JS ⟶ PA	0.566	8.278	< 0.001	Confirmed

*Note: N* = 385 (number of ICU nurses included in the analysis after excluding incomplete responses). PA: professional autonomy, measured by the Varjus Professional Autonomy Scale (V-PAS). JS: Job satisfaction, measured by the Minnesota Satisfaction Questionnaire (MSQ). OC: Organizational commitment, measured by the Porter Organizational Commitment Questionnaire (P-OCQ). Beta coefficient (*β*): standardized path coefficient indicating the strength and direction of the relationship between variables. *t*-value: test statistic for assessing the significance of the path coefficient (*t* > 1.96 indicates significance at *p* < 0.05). Significance (*p*): probability value indicating statistical significance (*p* < 0.05 considered significant).

**Table 8:** Indirect effects and bootstrapping results with mediation analysis for the main model.

Path	Indirect effect	Bootstrap standard errors	95% Bootstrapped confidence interval	
			Lower	Upper
Organizational commitment to professional autonomy through job satisfaction	0.2744	0.0808	0.1122	0.4306

*Note:* Indirect effect: the effect of the independent variable on the dependent variable through the mediator (job satisfaction). Bootstrap standard errors and 95% bootstrapped confidence interval: derived from 5000 bootstrap samples to assess the significance of the mediation effect. Confidence intervals excluding zero indicate significant mediation.

## Data Availability

The data that support the findings of this study are available on request from the corresponding author. The data are not publicly available due to privacy or ethical restrictions.
